# Paying SPECIAL consideration to the digital sharing of information during the COVID-19 pandemic and beyond

**DOI:** 10.3399/bjgpopen20X101072

**Published:** 2020-04-29

**Authors:** Laura Armitage, Beth K Lawson, Maxine E Whelan, Nikki Newhouse

**Affiliations:** 1 Clinical Researcher, Nuffield Department of Primary Care Health Sciences, University of Oxford, Oxford, UK; 2 Research Coordinator, Nuffield Department of Primary Care Health Sciences, University of Oxford, Oxford, UK; 3 Post-Doctoral Research Assistant, Nuffield Department of Primary Care Health Sciences, University of Oxford, Oxford, UK; 4 Post-Doctoral Research Fellow, Nuffield Department of Primary Care Health Sciences, University of Oxford, Oxford, UK

**Keywords:** Quality assurance, Infectious illness, Social media, Information dissemination, COVID-19, Severe acute respiratory syndrome coronavirus 2, Communication, Communications media, Primary health care

## Background

Within weeks of identification of COVID-19 disease in China, social media and digital information sharing platforms such as Twitter, Facebook, and WhatsApp saw the rapid spread of information, termed an ‘infodemic’ by the World Health Organisation (WHO).^1^ Some of this information has been high quality science while much more has been misleading, fear-mongering, or false. In some instances, the distinction between high quality and false information is less clear, but the information creates distraction or confusion. This can be harmful at a time when trustworthy science and guidance in the area of COVID-19 are urgently needed.^1^

Researchers and health professionals are among many sharing information using digital platforms and social media. Seeking to share knowledge, protect others from risk, and provide leadership may lead us to media-trawl for the latest information and feel an urgency to share what we discover. We too may contribute to spreading information that creates fear or leads to harmful outcomes. A number of researchers and health professionals have major influence as opinion leaders among the public and their peers. Primary care clinicians and researchers should promote a critical and sceptical approach to the information we share, and those in leadership positions have a particular responsibility to lead by example.

Social media and digital information platforms have major potential to help health services respond to COVID-19, by disseminating up-to-date and accurate advice.^2^ To achieve this, people must be able to access credible advice over misinformation. In 2009, the WHO published advice on the use of social media for disseminating health information; the bottom line is that we should be strategic and choose wisely.^3^

**Figure 1.**
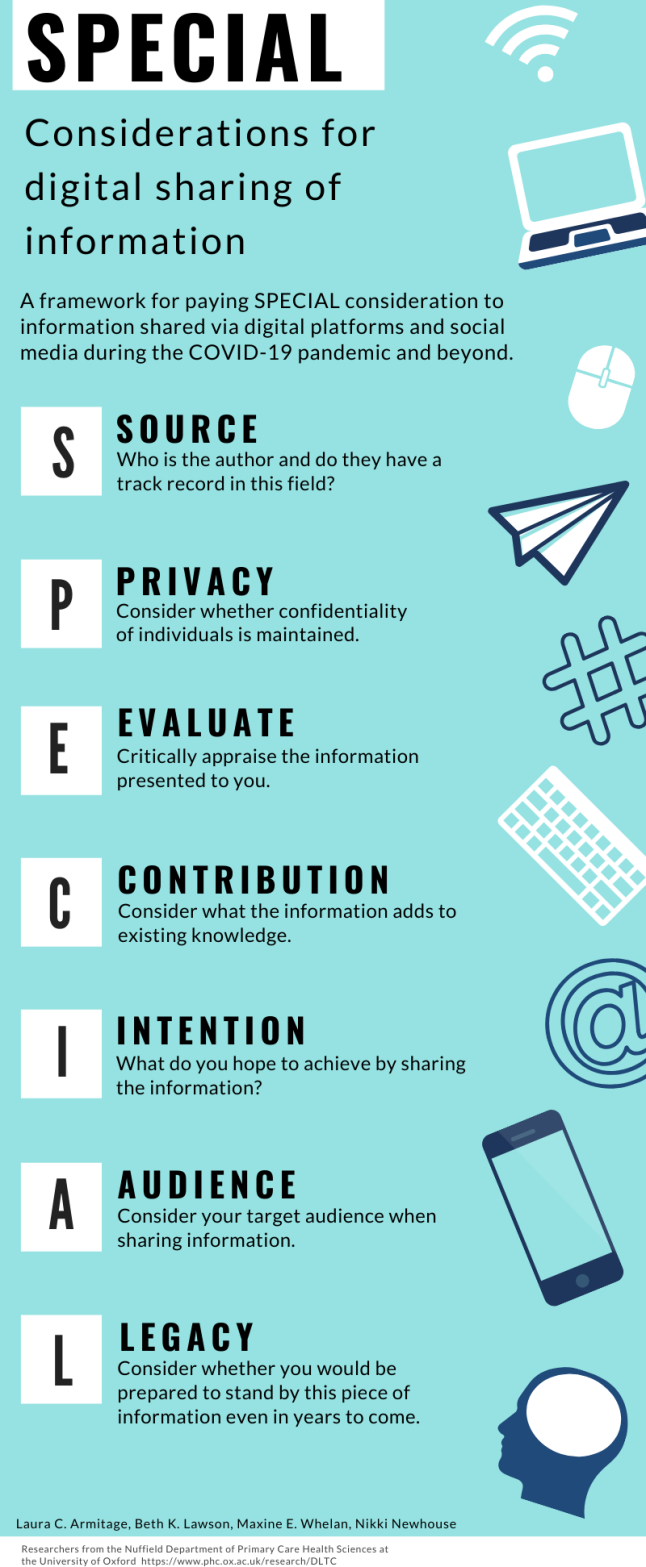
A framework for paying SPECIAL consideration to information shared via digital platforms and social media during the COVID-19 pandemic and beyond

We propose a framework to help us be strategic and choose wisely, by paying SPECIAL consideration to the information we share (Figure 1).

### S — SOURCE

Consider where the information has come from. Who is the author and do they have a track record of publishing or working in this field? If the information is a media article, social media post, photo, letter, or opinion piece, can you trace the original author? Before sharing, you should be comfortable that the author is credible and the information can be verified. If the information is a scientific article, pay attention to whether it is published in a reputable journal with clear peer review processes. If the author has published their work somewhere other than a peer reviewed journal, consider why that is. Barriers to rapid dissemination including long periods for peer review and publication fees have been dramatically reduced to support access to important new findings. If the article is published on an institutional website, look for evidence of internal peer review and consider the influence this may have. In all instances, funding sources for scientific research should be clearly stated, helping the reader consider potential sources of bias.^4^

If considering posting an original piece of information with yourself as the original source, take a moment to consider whether what you are sharing is evidence-based and helpful. Could your peers and community follow these steps, and feel safe and comfortable in sharing your information piece as a credible and trustworthy source of information?

### P - PRIVACY

Consider whether confidentiality of individuals referred to within the information is maintained. No identifiable information should be presented in scientific articles without explicit consent. All scientific medical articles should report ethics committee approval and consent procedures. If you are concerned an article breaches an individual’s confidentiality or serious study misconduct has occurred, you should raise your concern with the publishing journal’s editor.^5^

For media articles, social media posts, photos, or letters, review whether the identities of or information about individuals are revealed. If so, is there implicit or complicit consent from these individuals to share this information?

### E - EVALUATE

Proactively evaluate or critically appraise the information presented. How has the information been presented? Is it presenting a balanced argument?

There are four major questions to consider when critically appraising any scientific study: (1) did the study address a clearly focused question?; (2) did the study use valid methods to address the question?; (3) are the valid results of this study important?; and (4) are these valid, important results applicable to my patient or population?^6^ If the answer to any one of these questions is no, it would be appropriate to refrain from sharing the information. For media articles, photos, letters, and opinion pieces, consider what ‘evidence’ is in the piece. Is there transparency regarding where this information has come from? Is the context of the information appropriately interpreted and presented?

### C – CONTRIBUTION

Consider how the information adds to existing knowledge. Does it take other recent knowledge or developments into account? Does it replicate or challenge existing evidence? Does it help us consider the validity of existing knowledge? If it challenges existing knowledge and research, we recommend critically appraising the conflicting pieces of information and reviewing their risks of bias.

### I – INTENTION

What do you hope to achieve by sharing the information? Are you aiming to improve people’s understanding? Perhaps your aim is to improve patient care? Do you have a major concern about an area of practice or piece of research that you wish to quickly disseminate? If so, is this well-founded and evidence-based? In some cases, there may be a conscious or subconscious desire to increase one’s own presence, reach, or status on social media or online platforms. Sharing information with this underlying intent only may distract people from accessing the most important information.

### A – AUDIENCE

Consider *your* target audience when sharing information. There have been calls on social media from the public for healthcare professionals to refrain from posting information that scares the public. We do not suggest a blanket rule regarding this. Instead, it might be appropriate to consider who your audience is, whether your post may contribute to the generation of fear, and, if so, what is the most appropriate platform for sharing this particular information? If you decide to post a piece of information publicly on social media, can you stand by it if challenged? If you are raising a concern about current practices in your health service, are you prepared for this concern to be escalated with your name beside it? Have you considered other channels for raising concerns, and is sharing your concern on social media or a digital sharing platform necessary and proportionate?

### L – LEGACY

Consider the legacy that sharing this information may have and whether you are prepared to stand by it, even in years to come. We should all be mindful that anything we share digitally or post on social media is permanent. Even if deleted, social media posts are archived and can be traced. In addition, screenshots may be taken and disseminated on other platforms too, often without an individual’s knowledge or consent. Remember to attend to codes of professional conduct, being mindful of who and what you represent. This might include your profession, employer, family, and friends.

## Discussion

### ​Practical tips to reduce the dissemination of misinformation or poor quality information

The volume of information presented to you through social media and digital information platforms may feel overwhelming. In this instance, there are several quick tips that might help filter the quantity and quality of the information presented to you, enabling you take the time to appraise a higher proportion of what you see (Box 1). These may also serve to reduce the dissemination and reach of lower quality or inaccurate information.

Box 1 **Quick steps for filtering the quantity and quality of information presented to individuals via social media accounts and social platforms**

1. Identify quality sources of online information and bookmark these pages.
2. Consider which social media and online platforms you use and whether there are one or two that lend themselves to better quality information.
3. Think about whether you want to limit who you follow on social media, to a smaller group of trusted people or organisations who you know disseminate reliable and high quality information.
4. If you don’t want to ‘unfollow’ individuals or organisations on social media, consider temporarily hiding the posts of those who frequently share information that does not meet high quality standards.

The proposed framework aims to help achieve the best from social media and digital platforms, and reduce misinformation and fear that can arise when these are not used in the best way possible. When used in the most positive ways, social media and digital platforms streams can educate, inform, uplift, and connect people and communities.
