# Correction: Paying SPECIAL consideration to the digital sharing of information during the COVID-19 pandemic and beyond

**DOI:** 10.3399/bjgpopen20X101160

**Published:** 2020-12-17

In the Practice & Policy article by Armitage *et al.* Paying SPECIAL consideration to the digital sharing of information during the COVID-19 pandemic and beyond *BJGP Open* 2020; DOI: https://doi.org/10.3399/bjgpopen20X101072, the Funding section incorrectly stated that no funding had been received for the article. This section should have stated: “This research was supported by the National Institute for Health Research (NIHR) Oxford Biomedical Research Centre (BRC). The views expressed are those of the authors and not necessarily those of the NHS, the NIHR or the Department of Health.” We apologise for this error. The online version has been corrected.

